# Challenging traditional research: A synopsis of the National Research Collaborative Meeting (NRCM) in 2017

**DOI:** 10.1016/j.isjp.2019.03.001

**Published:** 2019-05-13

**Authors:** National Research Collaborative Meeting 2017 Organising Committee, J. Glasbey, D. Nepogodiev, S. Kamarajah, Y.L. Goh, G. Layton, S.C. McKay, J. Singh, Y. Sinha, R. Wilkin, D.E. Yeung, A. Bhangu, P. Singh, on behalf of the West Midlands Research Collaborative

**Keywords:** Research collaborative, Surgery, Surgical research, Collaborative meeting, Research meeting

## Abstract

**Introduction:**

The National Research Collaborative Meeting (NRCM) 2017 was jointly hosted between the West Midlands Research Collaborative (WMRC) and Student Audit and Research in Surgery (STARSurg) on 30th November 2017 in Birmingham. The NRCM 2017 theme was *‘Challenging Traditional Research’*.

**Methods:**

Narrative review, outlining key challenges and recommendations for trainee collaborative research groups across medical and surgical disciplines based on the core themes from the NRCM 2017 meeting.

**Results:**

Core themes of: (1) surgical oncology trials; (2) placebo-controlled surgical trials; (3) research funding; (4) medical student involvement in research; (5) emergency care; (6) patient and public involvement. Recommendations were made for planning future collaborative studies, based on these topic areas.

**Conclusions:**

The collaborative research model has demonstrated longevity and effectiveness in delivering high-quality, practice-changing research both within the NHS and internationally. Learning between groups and highlighting areas for interdisciplinary collaboration will drive a meaningful, patient-centred agenda for the future.

## 1 Introduction

The National Research Collaborative Meeting (NRCM) 2017 was jointly hosted between the West Midlands Research Collaborative (WMRC) and Student Audit and Research in Surgery (STARSurg). The meeting was held at the Marriot Hotel in Birmingham on Friday 30th November 2017, and welcomed over 260 attendees and speakers from a range of medical and surgical specialities and diverse stages of training, discussing their views and experiences of collaborative research. Trainee-led regional and national collaborative networks adopt a novel approach to research and audit by ‘crowdsourcing’ data collection through frontline clinicians, allowing for a larger number of patients to be included in less time, reducing research waste, and permitting greater generalisability than single-centre studies [1]. Trainees are ideally placed to perform research in this way as they rotate through hospitals during their training, have established communication networks, and require formalised evidence of research and audit for progression.

**Fig. 1:**
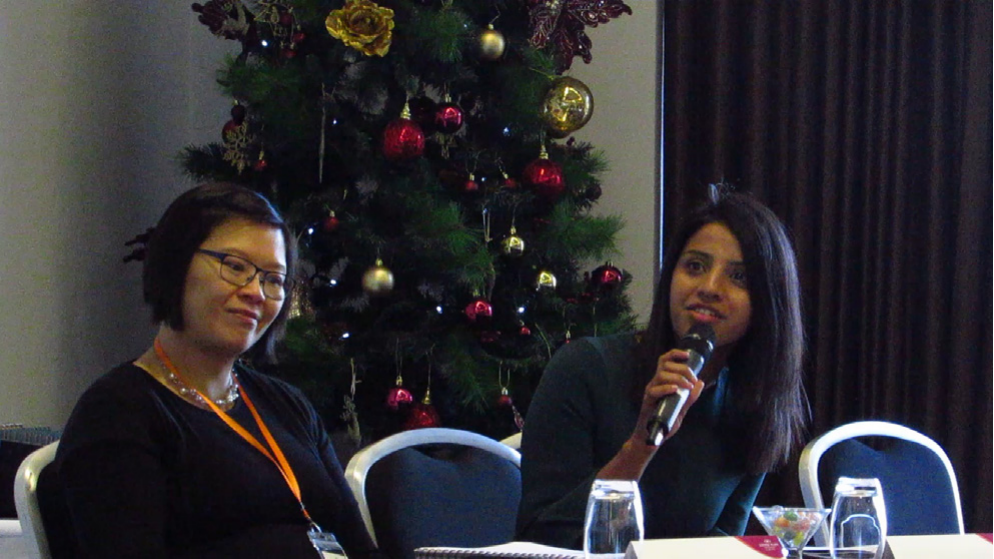
The National Research Collaborative Meeting 2017 panel members included interdisciplinary trainees, medical students, consultants and researcher leaders from around the world.

The collaborative research model has grown exponentially over the last 10 years, both in scope, delivering complex studies including randomised trials, and breadth, growing networks both nationally and internationally [2], [3], [4]. The core theme of the NRCM 2017 meeting was ‘Challenging traditional research’, highlighting both the many ways in which collaborative groups have broken down traditional hierarchies and delivery models and the hurdles groups have faced. Issues ranging from leadership of trials, student involvement, patient involvement, recruitment, funding, organisation to increasing complexity of trials have all been faced, with groups finding diverse solutions to overcome these Fig. 1.

**Fig. 2:**
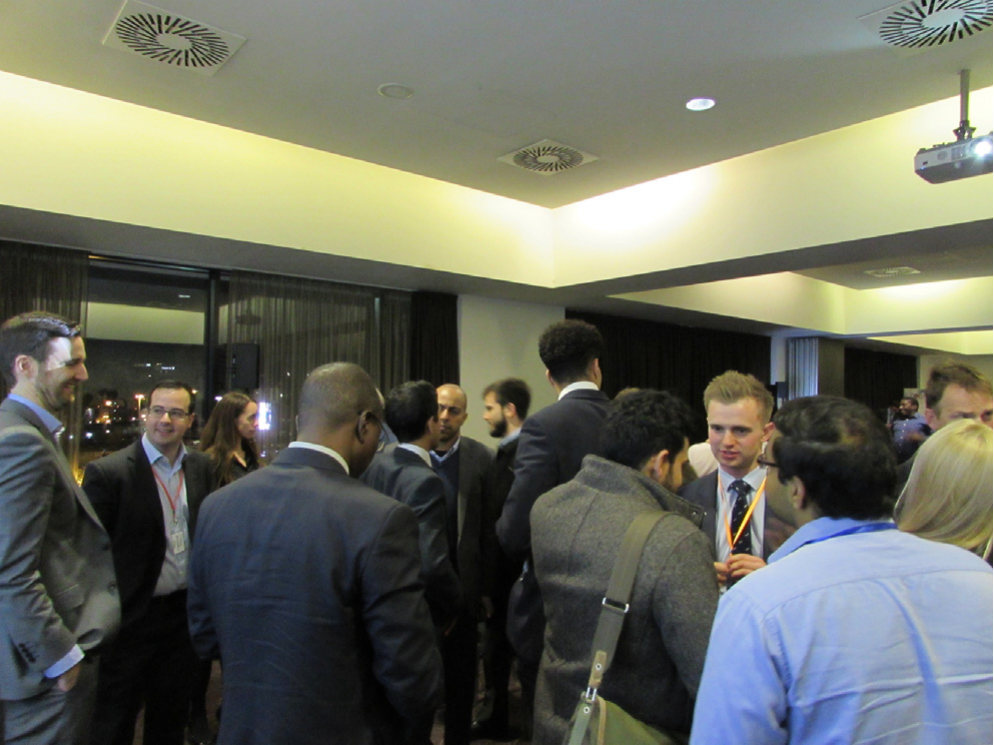
NRCM 2017 represented an opportunity for networking with colleagues and friends, and shared learning to benefit collaborative research worldwide.

NRCM 2017 brought individuals from across the UK and overseas to discuss their experience of overcoming these issues. Sharing barriers and solutions faced by other collaborative research groups can help achieve greater success for this research model in the future (Fig. 2). This editorial will summarise key areas of discussion and the learning points from this conference.

## 2 Meeting themes and learning points

### 2.1 Building trainees into surgical oncology trials

Clinical trials are considered the gold standard in evaluating healthcare interventions. Within national and international settings, surgical trainees have been empowered to and increasingly involved in multi-centre, prospective cohort studies [5], [6], [7]. The traditional challenges of clinical trials such as recruitment and describing uncertainty, standardisation and training of novel surgical interventions, and learning curve effects provides an extra layer of complexity to trainees wished to be involved in research [8], [9]. However, trainees can be supported in developing and leading clinical trials with access to senior trialists and the infrastructure from established clinical trials units (CTUs) through the research collaboratives [3].

Within surgical oncology, there is a growing infrastructure to support trainee-led trials in cancer through the Clinical Studies Group (CGS) initiative from the National Cancer Research Institute (NCRI). The CGS collaborates between patients, researchers, funders and other relevant networks to provide a framework for developing cancer clinical trial ideas in the UK. The NCRI along with other national bodies such as the Royal College of Surgeons facilitate workshops to develop cancer trial research skills. These workshops work on global development of trial research as well as focusing on integrating trials into a busy department, trial design, research nurses and the need for a trial’s unit. The STAR-TREC trial (Can we Save the rectum by watchful waiting or TransAnal microsurgery following Radiotherapy versus Total mesorectal excision for early REctal Cancer) was given as an example of a trial where trainee involvement has been essential to set-up and early delivery.

*Learning point 1:* Trainee research collaboratives should work with specialised clinical trials units and regional representatives from established supporting frameworks to lead and recruit to trials in surgical oncology.

### 2.2 Placebo-controlled surgical trials

Within the context of a traditional randomised drug trial, a novel intervention is compared against a placebo, whereby the latter is considered an ‘inert’ or non-active substance or component [10], [11]. However, in the surgical setting, the delivery of a ‘sham’ or non-therapeutic surgical procedure raises a number of complex ethical and logistical issues [12]. Some have argued that exposing a trial participant to a surgical procedure, with the inherent risks of general anaesthetic, infection, bleeding, and postoperative pain to name a few, is not equivalent to taking a placebo pill, and therefore inherently not ethical [13]. However, modern trialists have proposed that the risks involved in having an operative intervention itself are potentially too harmful and invasive to not be robustly tested with double-blind randomized placebo-controlled trials [11]. There have been few examples of such placebo-controlled surgical trials in the past. Relevant examples included injection of foetal dopamine neurons into the brains of patients with Parkinson’s disease [14] and arthroscopic debridement in arthritis of the knee [15]. The importance of placebo control in surgical trials was underlined specifically in Beard et al. (2018), which demonstrated arthroscopic subacromial decompression for subacromial shoulder pain not more effective than having an arthroscopy alone. Despite ethical and logistic issues, placebo-controlled surgical trials are likely to become increasingly important in generating future high quality clinical effectiveness evidence, when the condition of clinical equipoise is met. A 2018 MRC-NIHR funded workshop has begun to create a set of principles and guidelines to govern placebo-controlled trials which will be available to adopt into future studies [16].

*Learning point 2:* Trainee collaboratives considering a placebo-controlled surgical trial should consult a Clinical Trial Unit with specialist experience of managing these studies, and consider future consensus recommendations.

### 2.3 Funding for trainee collaborative studies

Funding for clinical trials traditionally comes from three sources: (i) industry, (ii) research bodies such as the NIHR and, (iii) charities with special interests such as Cancer Research UK [17]. Securing funding can be challenging but ensuring an impartial funding which will have no influence on reporting and interpretation of trial results is essential [18], [19]. Obtaining competitive research funding can seem difficult for even the most-experienced chief investigator. However, trainee-led randomised control trials can and do accrue funding, by conducting the correction preparations, including observational, and pilot and feasibility studies [4], [20].

Trainees with experience of developing trials and securing funding emphasised the importance of proof of concept to support any funding applications by conducting a robust primary feasibility and or pilot randomised study [21]. Feasibility studies answer specific questions about the study design and are typically non-randomised, for example delivery of a stable intervention, testing a follow-up pathway, or identifying an optimal primary outcome measure. Pilot studies are typically randomised and are designed as small, unpowered versions of main phase III trials, with different aims, objectives and outcome measures to main trials [22], [23]. This answers important questions about trial delivery, for example: (i) Can you recruit to the trial?, (ii) Can you train people to deliver an intervention?, (iii) Can you follow-up patients after discharge?, (iv) Can you get sufficient data completeness for your desired analyses [24]? This evidence then bolsters a later funding application. Furthermore, applications should be supported by a team with broad and varied experience, including health economists, trial methodologists, statisticians, patient and public (PPI) representatives and other specialised members dependent on the trial design. Funded, trainee-led randomised control trials are likely to become more frequent, especially in the wake of the increasing popularity of trainee-led research collaboratives. Procuring funding remains a rate-limiting step for all clinical trials but a robust evidence-based design and experienced support for funding applications will increase the likelihood of success.

*Learning point 3:* When seeking funding for a major interventional study, trainee research collaboratives should consider a pilot or feasibility study, contacting a Clinical Trials Unit early, engaging a multi-disciplinary team including PPI representatives and using data from observational collaborative studies to power and justify their study designs.

## 3 Student involvement in collaborative research groups

Traditionally, getting involved in research as a medical student can be difficult, in terms of finding the right project and a right supervisor or mentor to support students from study design to data collection, and eventually to publication. Historically, embedding medical students into research projects had been perceived to be challenging, with opponents quoting lack of understanding, poor data reliability, and lack of time for meaningful contributions. To overcome such barriers, the STARSurg collaborative was launched in 2013 and is the UK's only national student-driven collaborative with national representation across all medical schools in UK and Ireland. Since its inception, STARSurg has delivered four national cohort studies and has gained increasing participation amongst students, junior doctors and consultants [5], [6], [7], [25]. Importantly, all these studies have performed external, independent validation of case ascertainment (>95% overall) and data accuracy (>98% overall). The studies have also improved students’ knowledge of audit cycles, collecting data in a clinical setting and presenting results in a scientific manner to drive improvement in care quality [26]. The STARSurg collaborative provided a strong foundation for student participation in collaborative research, giving students a toolkit to take forwards into their postgraduate career [26]. Improving links with postgraduate research groups across specialties to maintain interest during early clinical years is a major national objective for the future.

*Learning point 4:* Students can deliver research with high levels of accuracy and case ascertainment, learning valuable skills for their future careers and bolstering postgraduate collaborative groups.

## 4 Research in the emergency setting

The busy emergency care setting could be perceived as an environment that is both hostile and challenging to undertake robust clinical research. Rapidly changing potential life-threatening clinical conditions, time-pressured decision-making with little time for significant deliberation during the consenting process, and unwell patients with decreased levels of consciousness are some of the many challenges faced by the researcher. This however is not an insurmountable barrier, evidenced by the success of the National Audit of Small Bowel Obstruction (NASBO), a trainee-led, national study with more than 460 collaborators from 131 centres [27]. NASBO captured data on 2434 cases, reflecting the high burden of disease, with a mortality of 7.8%, and identified key areas for future study, such as malnutrition being associated with a two times greater risk of death in their patient cohort.

Fluid Optimsation in Emergency Laparotomy (FLOELA) is a further example of high-quality emergency care research, a clinical trial investigating the effect of cardiac-output guided haemodynamic therapy during and shortly after emergency bowel surgery [28]. This trial demonstrates the integral role trainees have in successfully delivering emergency care research, as they provide frontline care out-of-hours, therefore have immediate access to recruiting and consenting patients, and collecting data. Excitingly FLOELA has embraced and acknowledged the key role the trainee plays in these studies, having trainee associate principle investigators from surgery and anaesthetics for each site, a model to be emulated in future studies.

*Learning point 5:* Trainees are invaluable in recruitment to emergency care studies within their role of delivery of frontline care. Particular consideration of emergency care topics should be made by research collaboratives when planning future studies.

### 4.1 Authorship models within collaborative research

Over the past decade, it has been recognised that national and international collaborative research allows clinical questions to be addressed in a meaningful way by collecting high volume data from multiple centres. The contribution of many members of large complex teams across specialties, countries and disciplines requires authorship structures which credit different roles sufficiently and give clarity in terms of task involvement. The National Research Collaborative and UK Association of Surgeons in Training have created a draft framework for collaborative research authorship groups, which delineate roles including a writing group, steering group, statistical analysis, REDCap and data governance, national, regional and local leads, collaborators, validators and translators [29]. A more detailed outline for groups wishing to use a full corporate authorship model can be found at: http://www.wmresearch.org.uk/. Collaborative authorship is now recognised at trainee-level for selection and progression at ARCP, and is acceptable as part of Certificate Completion of Training (CCT) criteria in a number of specialties.

*Learning point 5:* Clarity in terms of contributions of large numbers of collaborators in collaborative research studies can be ensured by adherence to published frameworks. Transparency from the offset about the intended authorship model is crucial to maintain networks and avoid disincentivising collaboration.

### 4.2 Embedding PPI into collaborative studies

Patient and public involvement (PPI) in research is recognised as best practice and is now an essential requirement to receive funding from many funders globally, including the UK, the Netherlands, Canada, Australia and the USA [30]. Indeed, the UK National Institute for Health Research (NIHR) supports the INVOLVE programme, established in 1996 to support active public involvement in healthcare, public health and social care research. INVOLVE aims to advance the role of PPI in all aspects of the research process, including research prioritisation, design, conduct and dissemination [31], [32].

Patient involvement has been proven to improve key trial processes such as recruitment and retention, and make design and outcome assessment more patient relevant [33], [34]. Observational research studies steering groups are also making efforts to improve patient representation both within their study planning, steering and reporting.

*Learning point 6:* Patient and public involvement is crucial for all study types and should be considered at all points in the study pathway. Good PPI within a study will make it more meaningful, impactful and fundable.

## 5 Conclusion

Within modern clinical research in the UK traditional ‘top-down’ research models have been replaced with ‘bottom-up’ trainee-led research collaborative projects. Proof-of-concept studies have demonstrated that trainees and students can play a key role in practice-changing research and are capable to leading high-quality cohort studies and randomised trials with support and guidance from senior members. Early engagement and mentoring of students and trainees alike will drive a new cadre of academic surgeons and NHS surgeons with strong academic interests in clinical research. The NRCM 2017 meeting reflects that successful research in the modern age, lies in collaboration of different groups to improve and deliver high-quality care to patients.

## NRCM 2017 Organising Committee

Glasbey J^1^, Nepogodiev D^1^, Kamarajah S, Goh YL, Layton G, McKay SC, Singh J, Sinha Y, Wilkin R, Yeung DE, Bhangu A, Singh P

^1^Meeting co-leads
